# Naked‐Eye Visual Warning of ppm_v_‐Level Fault‐Free Humidity Upper Limit

**DOI:** 10.1002/advs.202518311

**Published:** 2026-01-29

**Authors:** Xiaoyan Wei, Tianyou Qin, Siyu Wang, Sean Xiao‐An Zhang, Lan Sheng

**Affiliations:** ^1^ State Key Lab of Supramolecular Structure and Materials College of Chemistry Jilin University Changchun P. R. China; ^2^ Department of Biochemistry and Molecular Biology College of Basic Medicine Science Jilin University Changchun P. R. China

**Keywords:** colorimetric humidity sensing, humidity detection, hydrochromic molecular switches, naked‐eye detection

## Abstract

Monitoring and warning of ppm_v_‐level fault‐free humidity upper limit (ppm_v_‐FFHUL) are crucial for ensuring system reliability in critical fields such as high‐tech manufacturing, and aerospace. However, existing ppm_v_‐FFHUL warning technologies are scarce, and constrained by “gradual change” response modes, resulting in complicated equipment, high costs, and insufficient portability, which greatly limits their flexible application. To address this, we propose a “leap change” response mode and an “enzyme‐like” construction method for precise ppm_v_‐level humidity threshold modulation. It is achieved through synergistic supramolecular interactions between two size‐matched auxiliary mediums to precisely construct an “enzyme like” microenvironment containing a matching number of multi‐level, spatially fixed sensitizing functional groups around an elaborately designed humidity‐sensitive component. The as‐prepared material combines paper‐like flexibility, facile fabrication and handling, and enables high‐contrast readout of the target ppm_v_‐FFHUL only by naked‐eye. Moreover, it holds promise as an easily deployable ppm_v_‐FFHUL warning labels and provide a visual pre‐alert signal 20% ahead of the critical value. It offers a portable and low‐cost solution for specialized scenarios that require early warning and rapid screening of ppm_v_‐level humidity. Furthermore, the “enzyme‐like” precise construction method and the “leap change” response mode provide an innovative perspective for the design of other high‐performance trace‐level monitoring materials.

## Graphical abstract

In this study, based on the urgent need in many critical fields for ppm_v_‐level fault‐free humidity upper limit (ppm_v_‐FFHUL) monitoring, an innovative threshold gated humidity sensing technology was developed. It exhibits superior portability (as thin and light as paper) and highly economic feature (without auxiliary instruments at all), and can achieve real‐time monitoring and efficient early warning of ppm_v_‐FFHUL.

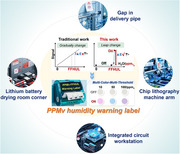

## 1 Introduction

Monitoring and warning of ultra‐low humidity (≤100 ppm_v_ (i.e., parts per million in volume)) play a crucial role in key fields such as high‐tech manufacturing [1, 2, 3, 4, 5] (e.g., chip packaging, solar cells, lithium‐ion batteries), power transmission [6, 7], national defense and military industries [8], and aerospace exploration [9]. The significance of ppm_v_‐humidity warnings is further disclosed by statistical analysis, which reveals that approximately one quarter of global industrial manufacturing defective and discarded products each year can be attributed to the failure to provide timely warning against excess environmental moisture, resulting in enormous and immeasurable economic losses. Theoretically, the lower the humidity in the production environment, the more beneficial it is for ensuring product quality. While as humidity decreases, the corresponding moisture control costs increase exponentially. Therefore, in order to reasonably control costs and maximize production benefits, industries with ultra‐low humidity control requirements have implemented the fault‐free humidity upper limit (FFHUL) warning strategy. FFHUL refers to the highest humidity threshold required for products to be manufactured without faults and smoothly while ensuring optimal cost efficiency. Exceeding this critical threshold would significantly amplify the probability of product malfunction [4, 10].

**SCHEME 1:**
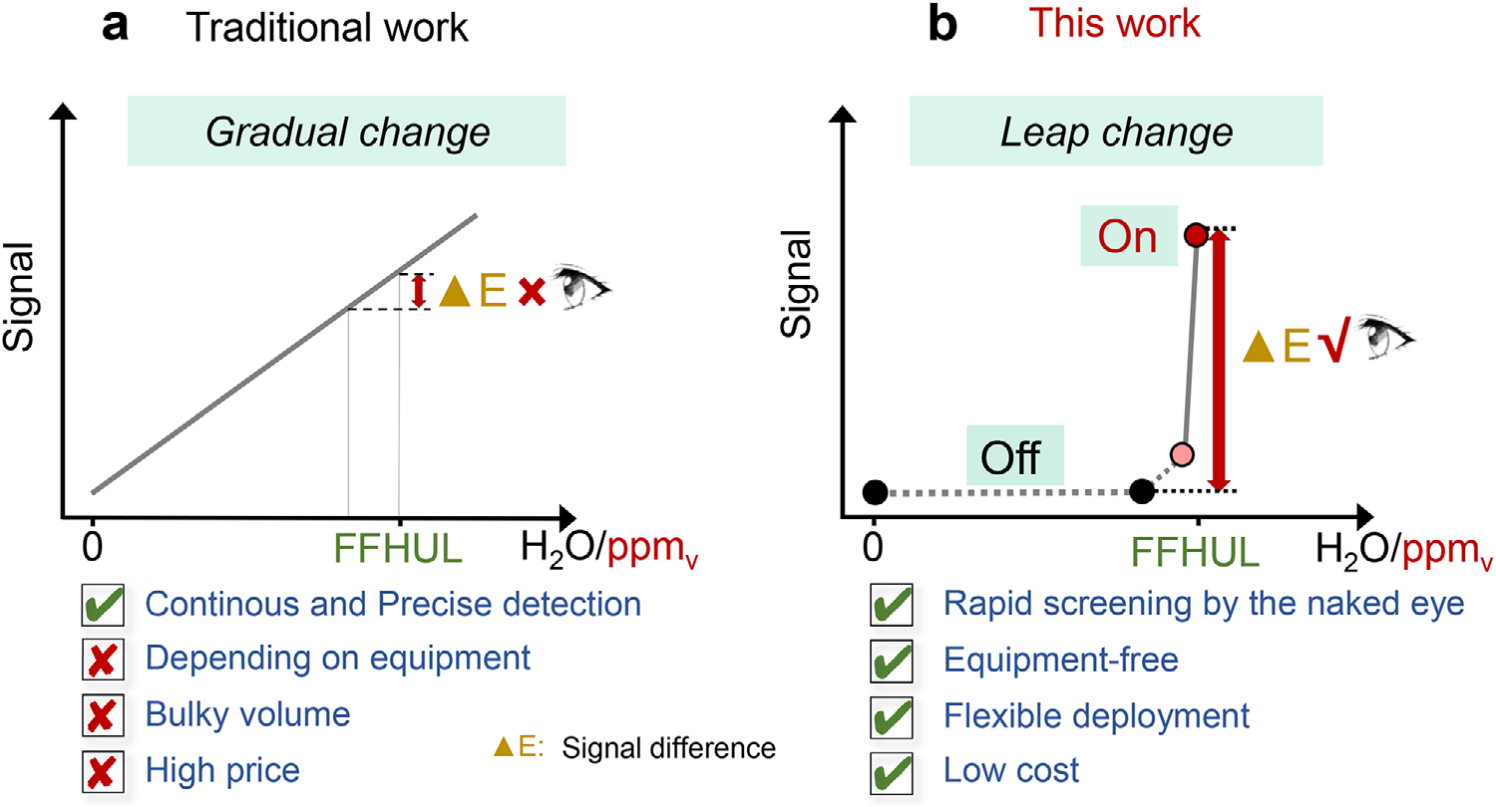
(a) Traditional and (b) this work of the warning material working mechanism differences.

Notably, given the extremely low abundance of water molecules in ppm_v_‐level humidity environments, ultrahigh sensitivity is indispensable for humidity sensors. Consequently, there are very few technologies currently capable of providing ppm_v_‐level FFHUL warnings. The currently commercial ppm_v_ humidity detection technologies rely on capacitive humidity sensors based on γ‐alumina with specific crystalline phases and moisture dew point meters (e.g., Michell Easidew, Shaw, Xentaur LPDT, GE Panametrics M series, and ZDC Tech E100) [11, 12, 13, 14]. These instruments play a crucial role in scenarios that require continuous, rapid, and accurate detection of ppm_v_ humidity. However, the demands for high precision and facile signal readout—which necessitate the assistance of external circuits, signal conversion, and display devices—have led to a trade‐off and compromise in sensor cost and size, thereby restricting their flexible deployment in certain scenarios (Scheme 1a). In recent years, visual sensing materials based on supramolecular systems/interactions (e.g., test strips, smart hydrogels) [15, 16, 17, 18, 19, 20], leveraging their significant advantages such as portability, strong scenario adaptability, ease of operation, and controllable cost, have provided novel insights for humidity detection. However, the humidity response of these visual materials typically exhibits a “gradual response” mode, where the signal often manifests as a continuous gradient change of a single color from light to dark. Therefore, it is difficult to distinguish the readings of the specified humidity threshold by the naked eye, and spectral instruments are still required for this purpose.

If the conventional “gradual change” response mode can be improved to produce a “leap change” response mode that triggers a distinct color change from colorless (OFF) to colored (ON) at a specific ppm_v_‐level humidity threshold, this pronounced chromatic transition would significantly enhance unaided visual detection (Scheme 1b), thereby contributing the development of portable ppm_v_‐FFHUL warning materials. However, achieving such a precise, rapid, and “leap change” response to specific humidity thresholds in ppm_v_‐level humidity environments remains highly challenging. This stems from three primary obstacles: first, breakthroughs in molecular/material design are required to develop a water‐induced colorimetric system with nonlinear response characteristics. This would shift away from the conventional gradual color change mode, which shows a color gradient with increase of switch molecules in the response state. Instead, a new mechanism must be established—where these molecules remain inactive at the initial stage and undergo a color change only when water molecules reach a specific quantity or threshold. Secondly, ultrasensitive molecular recognition capability is necessary to ensure a leap transition from the colorless to colored state within just a few ppm_v_ of water molecules. Lastly and most importantly, this color change must exhibit high contrast to ensure unambiguous discrimination by the naked‐eye.

From the knowledge of biochemical cell signal transduction, we understand that protein kinase A needs to bind to a sufficient amount of cyclic adenosine monophosphate (cAMP) to be activated and release catalytic subunits. This activation mechanism similar to a threshold switch is likely attributable to the intricate, spatially fixed multi‐level architecture of protein kinase A, which facilitates its interactions with cAMP and enables precise, quantifiable, and highly efficient activation in the auxiliary microenvironments. Inspired by that, we propose an “enzyme‐like” precise construction strategy—precisely engineering a multi‐level microenvironment incorporating a designated number of spatially fixed sensitizing functional groups around a humidity‐sensitive component at molecular level—may be an effective way to realize precise, rapid and “leap change” response to specific ppm_v_‐level humidity.

As a proof‐of‐concept, we design a high color contrast hydrochromic molecular switch as molecular‐level humidity‐sensitive component and the precise construction of multi‐level spatially fixed sensitizing microenvironment with matching number of hydroxyl groups around it. The obtained material exhibits a “leapfrog change” response to a specified ppm_v_‐level humidity threshold and can be high‐contrast readout of ppm_v_‐FFHUL value by the naked‐eye without requiring any signal conversion devices. Such materials not only feature simple preparation and rapid response speed (visually detectable within 5 min), but also can provide early warning signals ahead 20% before the designated FFHUL threshold. Owing to its superior portability and deployment flexibility, this material is expected to serve as a paper‐like humidity alarm label, providing an economical and convenient technical solution for related fields with the need for ppm_v_‐level humidity early warning, and rapid screening. This work has promoted the evolution of ppm_v_‐level humidity detection technology toward low cost, visualization, and easy deployment, and also offers new insights for the visualization and diversified development of trace detection technologies.

## 2 Results and Discussion

Although a variety of humidity sensors have been reported [21, 22, 23, 24, 25, 26], their applications in ppm_v_‐level humidity sensing are significantly limited because their humidity‐sensitive components typically respond to water through bulk‐phase changes or molecular aggregation behaviors rather than at the molecular level. Recently, indolino [2,1‐b] oxazolidine‐based hydrochromic molecular switches have been reported as molecular‐level humidity‐sensitive components. These switches rely on water (or humidity) induced cleavage of the bridge carbon (C)‐oxygen (O) bond in the oxazolidine ring to trigger a transition from a colorless ring‐closed form (RCF) to a conjugated zwitterionic ring‐open form (ROF) [27], which efficiently converts invisible humidity information into a visually perceptible color or optical signal. In our previous work [20], we pioneered the construction of a spatially fixed, polyhydroxy microenvironment around a nitro‐oxazolidine molecular switch (NO_2_‐OX), achieving continuous humidity response in the range of 0.01–200 ppm_v_, accompanied by a gradual color transition from light blue to dark blue.

**FIGURE 1:**
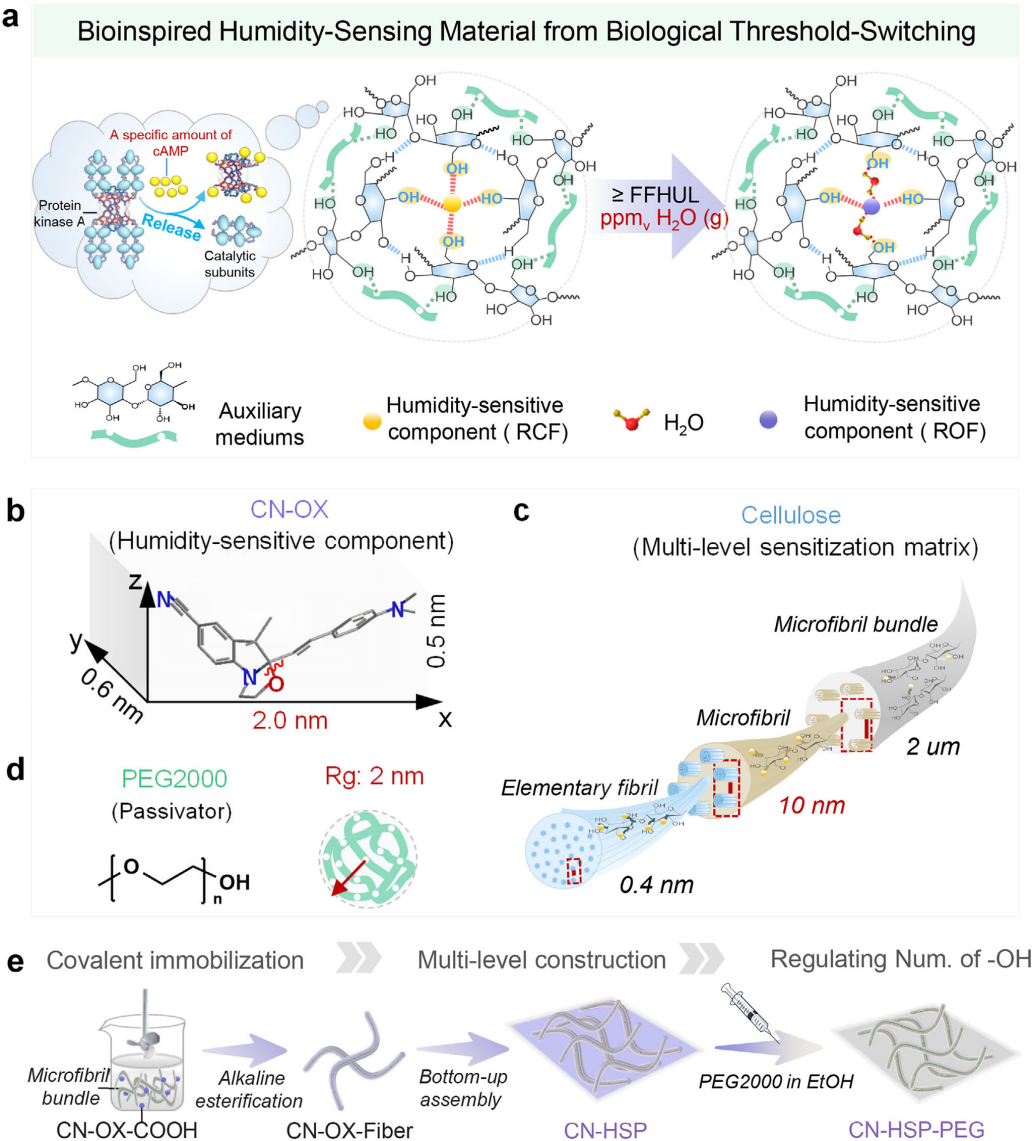
Design and preparation of materials. (a) Schematic diagram of constructing “enzyme‐like” multi‐level sensitizing functional groups with precisely controlled quantities around the molecular‐level humidity‐sensitive component. (b) Size of CN‐OX. (c) Micro‐nano pore size of cellulose network with multi‐level structure. (d) Radius of gyration of PEG2000. (e) Schematic illustration of the preparation process of CN‐HSP‐PEG.

Unfortunately, due to the contrast limitation caused by the color gradient response mode and the intrinsic absorption range, the specific humidity threshold cannot be identified by the naked eye, and its response speed is relatively slow (>15 min). Inspired by the results of this work and the multi‐level architecture and working mechanism of protein kinase A, we conclude that by designing a suitable hydrochromic molecular switch as molecular level humidity sensitive component, and precisely constructing an “enzyme‐like” multi‐level spatially assisted microenvironment with matching number of hydroxyl groups around it could enable a “leap change” visual response to specific ppm_v_‐level humidity thresholds (Figure 1a). Herein, we take 100 ppm_v_‐FFHUL, which is currently widely needed in practical applications, as an illustration.

Considering the structure of hydrochromic molecular switches, especially the substituents on the indolino‐part, can influence their water‐responsive behavior [28], this work engineered a cyano‐substituted oxazolidine molecular switch (CN‐OX) as the molecular‐level humidity‐sensitive colorimetric component (Figure 1b). This choice is based on two main reasons: 1) Compared with nitro group, the cyano group have weaker electron withdrawing effects [29], which may reduce the polarity of the C‐O bond and promote its ring‐opening isomerization, thereby potentially improving the response speed of CN‐OX to ppm_v_‐level humidity. 2) From an optical perspective, compared to the blue color exhibited by the ROF of NO_2_‐OX, the ROF of CN‐OX molecule shows a blueshift in its spectrum, leading to enhanced transmittance/reflectivity of red light. This color change lies within the optimal perception range of long‐wavelength sensitive cones in the human visual system, thereby improving the visual recognizability of the FFHUL warning.

Cellulose possesses a naturally occurring spatially fixed, multi‐level hydroxyl network [30, 31, 32, 33] (Figure 1c), combined with advantages such as environmental friendliness, availability, low cost, self‐supporting nature, and ease of processing, making it an idal carrier for construing a polyhydroxyl microenvironment. The challenge lies in precisely tuning the number of sensitizing hydroxyl groups to match CN‐OX and achieve rapid, “leap” response at 100 ppm_v_ humidity. Traditional methods foradjusting hydroxyl content through chemical modification (e.g., alkylation of primary hydroxyls) are not only cumbersome but may also hinder the grafting of CN‐OX.

In view of this, we propose a simpler and more efficient regulatory method. It achieves precise and controllable regulation of the number of spatially fixed hydroxyl groups by introducing a passivating matrix/reagent (i.e., hydrogen‐bond acceptor) and utilizing supramolecular interactions (i.e., hydrogen bonding) that match the size of the sensitizing matrix (i.e., cellulose). Based on size analysis, CN‐OX with a maximum radial dimension smaller than 2 nm can infiltrate the elementary fibril level of cellulose [34, 35, 36] (Figure 1b,c) and be sensitized by the spatially fixed polyhydroxy groups. Therefore, polyethylene glycol 2000 (PEG2000), which has a comparable size [37] (radius of gyration (Rg) ≈ 2 nm) and is rich in oxygen atoms with lone‐pair electrons, was selected as the passivator (Figure 1d and Equation S1).

To implement this strategy, the procedure involved first esterifying the carboxylated CN‐OX precursor (CN‐OX‐COOH) with pretreated filter paper microfibers to achieve fixation (CN‐OX‐Fiber). This was followed by a bottom‐up self‐assembly process and vacuum filtration under nitrogen protection to form the paper‐like structure (CN‐HSP). Subsequently, PEG2000 was introduced in a controlled and quantitative manner, utilizing the capillary action of the micro/nano‐scale pores in the fibers to reach the elementary fibril level, thereby enabling precise regulation of the number of spatially fixed hydroxyl groups surrounding CN‐OX (Figure 1e).

**FIGURE 2:**
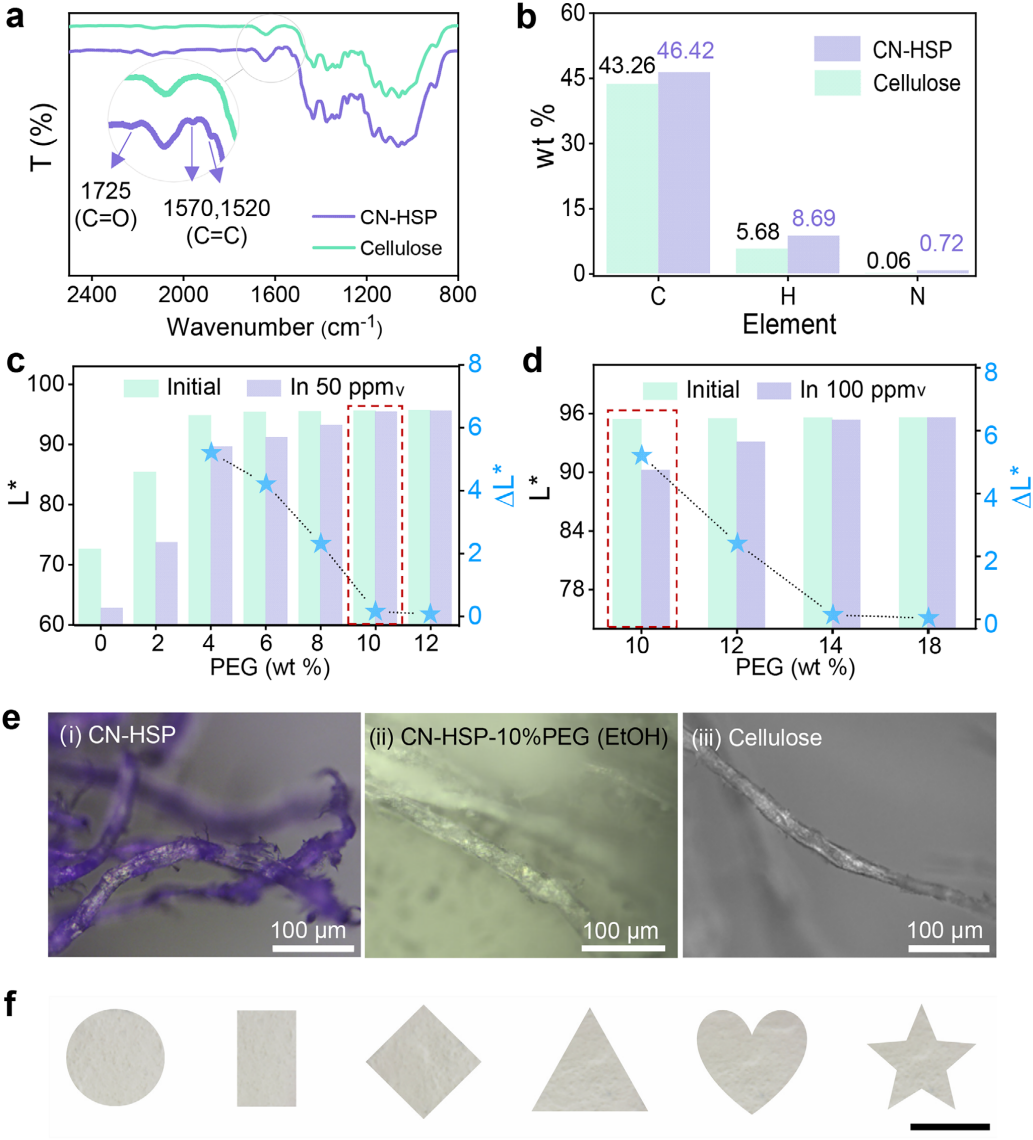
Characterization and optimization of passivator content. (a) FT‐IR spectra and (b) elemental analysis results of CN‐HSP and Cellulose. L^*^ value changes of CN‐HSP‐PEG with different PEG2000 contents in (c) 50, (d) 100 ppm_v_ moisture environments. (e) Optical microscope images of (i) CN‐HSP, (ii) CN‐HSP‐PEG constructed using ethyl alcohol (EtOH) as the permeation solvent, (iii) Cellulose. (f) CN‐HSP‐10% PEG cut into various shapes, scale bar = 1 cm.

The Fourier transform infrared (FT‐IR) spectroscopy (Figure 2a) indicate that CN‐HSP exhibit distinct spectral changes compared to unmodified cellulose. Specifically, a characteristic ester carbonyl (C═O) stretching vibration peak emerges at 1725 cm^−1^, along with two additional peaks at 1570 and 1520 cm^−1^, corresponding to the stretching vibrations of benzene ring carbon‐carbon double bonds (C═C). These results confirm the successful grafting of CN‐OX onto the cellulose backbone via esterification. Furthermore, elemental analysis of the contents of carbon, hydrogen, and nitrogen enabled an estimation of the CN‐OX grafting ratio, which was determined to be approximately 0.03 (Figure 2b, Table S1 and Equation S2).

The content of PEG2000 is critical for regulating the humidity response threshold of CN‐HSP‐PEG to FFHUL‐100 ppm_v_. At first, 50 ppm_v_ humidity is selected as the initial screening condition, and CN‐HSP‐PEG needs to remain colorless before and after responding to this humidity to ensure that it is in a stable OFF state before responding to 100 ppm_v_. As shown in Figure 2c, in the absence of PEG (0 wt.%, i.e., CN‐HSP), its chromatic value L^*^ (L^*^ value reflects color brightness, where lower values indicate deeper colors and higher values indicate lighter colors, approaching white at L^*^ = 100) was measured at 72 under initial conditions (gray bar). This suggests that the CN‐OX units in CN‐HSP is mainly in the colored ROF state. With increasing PEG2000 content from 2 to 12 wt.%, the L^*^ value of CN‐HSP‐PEG in the initial state gradually increased to 96 and tended to be stable (gray bars), indicating that the introduction of PEG promoted the transition of CN‐OX units from ROF to RCF. However, when the PEG content was ≤ 8 wt.%, CN‐HSP‐PEG exhibited color response in advance at 50 ppm_v_, showing a decrease in L^*^ value (purple bars). In contrast, when the PEG content was ≥10 wt.%, no response was observed at 50 ppm_v_ (*ΔL* ^*^ = 0). At 100 ppm_v_, with the gradual increase of PEG content (from 10 to 14 wt.%), the *L* ^*^ value after the response also gradually increased (Figure 2d, purple bars), and the corresponding *ΔL* ^*^ gradually decreased, indicating that the response contrast would gradually decrease. When the PEG content was further increased (18 wt.%), the *ΔL* ^*^ value was almost zero, suggesting that it did not respond to 100 ppm_v_ humidity. Corresponding photographs can also intuitively confirm this point (Figure S1). Therefore, the optimal PEG2000 content was determined to be 10 wt.% in order to ensure the reliability of the material before the response and to produce a high contrast for naked‐eye observation.

Optical microscopy (Figure 2e) showed that in the absence of PEG, CN‐HSP exhibited the characteristic bright purple coloration of the CN‐OX (i), confirming the successful incorporation of CN‐OX and that it was in the ROF state before responding to humidity because of the “sensitization” effect caused by the excessive number of hydroxyl groups provided by cellulose. In stark contrast, upon the introduction of 10 wt.% PEG (CN‐HSP‐10% PEG), it appeared colorless, similar to the ungrafted cellulose microfiber (ii vs. iii), This observation further confirmed the successful penetration of PEG2000 into the cellulose pores near the CN‐OX units, effectively exerting the intended “passivating” role and switching CN‐OX to its colorless RCF state.

It is worth mentioning that the choice of solvent also plays a very important role in the successful introduction of PEG2000. We chose highly polar, hydroxy‐containing EtOH as the dissolution solvent, because it has a high degree of “similar affinity” with the hydroxyl group of cellulose. The multiple supramolecular interactions between ethanol and cellulose (e.g., hydrogen bonding, dipole‐dipole interactions) contribute to the effective infiltration of PEG2000 into cellulose fibrils and around CN‐OX units through capillarization. As a control, when tetrahydrofuran with low polarity and no hydroxyl group was used as the solvent, PEG2000 only stayed on the surface of cellulose microfiber bundles under the same conditions, making the microfibers still appear purple (Figure S2), indicating that PEG2000 failed to enter the interior of the microfiber and thus could not play a passivation role. As shown in Figure 2f, the obtained CN‐HSP‐10% PEG is similar to conventional filter paper and can be customized into various shapes of different sizes according to needs, which has excellent portability and flexible deployment.

**FIGURE 3:**
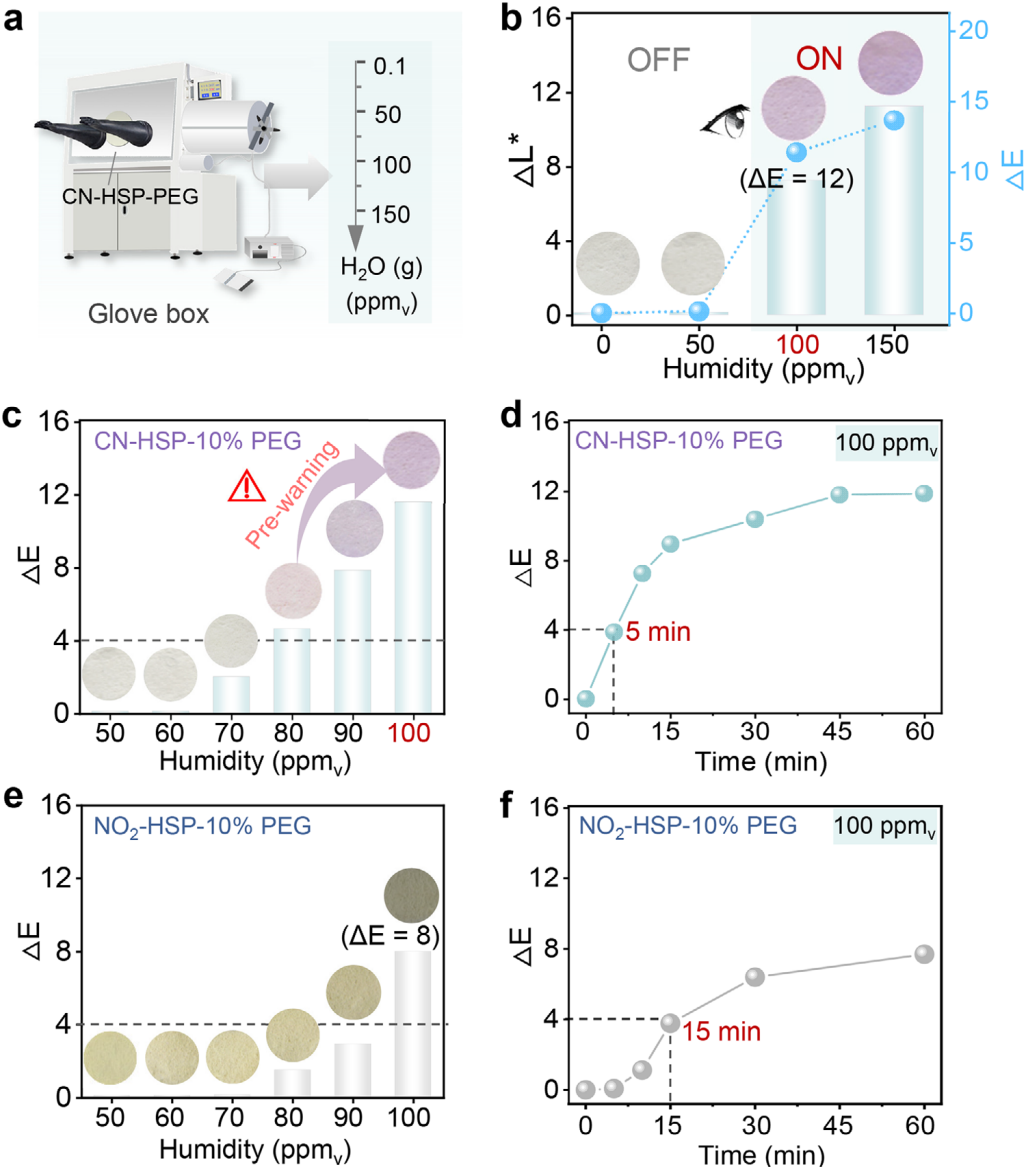
FFHUL Humidity Warning Performance. (a) Construction of humidity environments at different ppm_v_‐levels using a glove box. (b) Changes in ΔL^*^ and *ΔE* for CN‐HSP‐10% PEG at 0.1, 50, 100, 150 ppm_v_. (c) Changes in *ΔE* and corresponding optical photos for CN‐HSP‐10% PEG at 50, 60, 70, 80, 90, 100 ppm_v_. (d) Changes in *ΔE* over time for CN‐HSP‐10% PEG in a 100 ppm_v_ environment. (e) Changes in *ΔE* and corresponding optical photos for NO_2_‐HSP‐10% PEG in the range of 50–100 ppm_v_. (f) Changes in *ΔE* over time for NO_2_‐HSP‐10% PEG in a 100 ppm_v_ environment.

Subsequently, we evaluated the feasibility and related performance of CN‐HSP‐10% PEG as a visual warning system for 100 ppm_v_ humidity. A humidity‐controlled glove box was used to establish environments with varying ppm_v_ humidity levels, allowing real‐time monitoring of the response behavior of CN‐HSP‐10% PEG under corresponding humidity conditions (Figure 3a). Preliminary tests were conducted at 0.1, 50, 100, and 150 ppm_v_ humidity levels (Figure 3b). The inset image shows that CN‐HSP‐10% PEG remained colorless in environments below 100 ppm_v_, indicating it is in a stable “OFF” state. However, upon reaching 100 ppm_v_ or higher, it rapidly changes from colorless to deep purple, marking the transition to “ON” state. The change curve of *ΔL* ^*^ with humidity also reflected that the *ΔL* ^*^ value of CN‐HSP‐10% PEG remained unchanged less than 100 ppm_v_ humidity, whereas at 100 ppm_v_ and above, a significant leap occurred (green bars). These experiments show that CN‐HSP‐10% PEG can respond to a preset threshold‐100 ppm_v_ humidity with significant “OFF‐ON” signal switching. It is worth mentioning that the color difference value (*ΔE*) of CN‐HSP‐10% PEG before and after 100 ppm_v_ humidity response is as high as 12 (blue line plot), which means that this color can be easily perceived by the human eye (*ΔE* ≥4 can be observed by human eye) (Table S2 and Equation S3). It indicates that CN‐HSP‐10% PEG can obviously observe FFHUL‐100 ppm_v_ with high contrast through the naked eye.

In practical application scenarios, the ideal material capable of providing an early warning signal when the humidity approaches the preset critical threshold, thereby grants operators a time window for timely intervention, preventing significant losses to production caused by further increases in humidity. To further investigate whether CN‐HSP‐10% PEG has the warning function, we further refined the humidity intervals between the 50–100 ppm_v_ (Figure 3c). The results reveal that CN‐HSP‐10% PEG produces a slight color change at 80 ppm_v_, and its *ΔE* value is 4.5, which is just recognizable to human eyes. This indicates that CN‐HSP‐10% PEG can issue a preliminary warning before 20% of FFHU‐100 ppm_v_, and exhibits a great early warning ability. Besides, CN‐HSP‐10% PEG exhibits a relatively fast response speed, and can produce color changes that can be recognized by human eyes in about 5 min, so as to issue warnings in time (Figure 3d). In contrast, although NO_2_‐HSP‐10% PEG can respond to 100 ppm_v_ in the same situation, it not only has low contrast (*ΔE* is only 8), but also has no early warning function (Figure 3e), and its response speed is three times slower than that of CN‐HSP‐PEG (at least about 15 min) (Figure 3f). These above results highlight the critical role of the CN‐OX molecular switch in endowing CN‐HSP‐10% PEG with early‐warning functionality, rapid response, and superior visual warning performance.

Furthermore, we systematically investigated key performances closely associated with the future application of CN‐HSP‐10% PEG, such as gas selectivity, storage stability and reproducibility. In the gas selectivity tests involving common environmental gases like nitrogen (N_2_), oxygen (O_2_), carbon dioxide (CO_2_) and water (H_2_O), the material exhibited a specific response only to H_2_O, with no significant cross‐sensitivity observed, fully confirming its high selectivity toward H_2_O (Figure S3). Additionally, after being stored in the dark environment for 30 days, the material showed no significant degradation in humidity‐responsive performance (including response color contrast and response speed) (Figure S4), indicating good short‐term stability and meeting the practical requirements for storage and transportation as a disposable indicator label. Moreover, the material features a relatively simple preparation process, endowing it with excellent reproducibility. (Figure S5).

**FIGURE 4:**
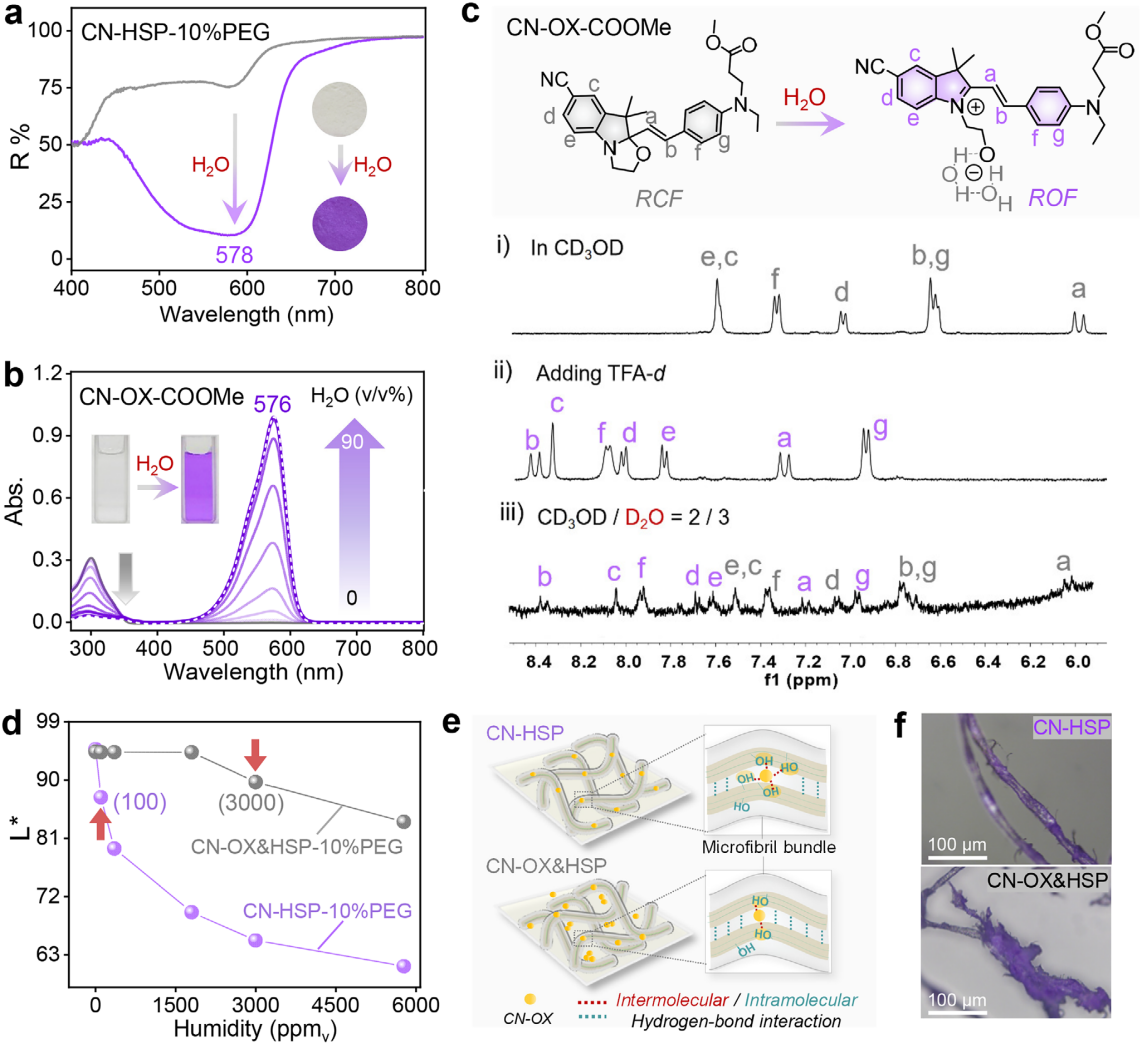
Naked‐Eye Visualization Mechanism of ppm_v_‐FFHUL. (a) The UV–vis reflective spectra of the CN‐HSP‐10% PEG before (gray trace) and after (purple trace) addition of water; Inset: photographs of the initial state and after addition of water of the CN‐HSP. (b) UV–vis absorption spectra of CN‐OX‐COOMe in acetonitrile upon addition of excess HCl and variable mixtures of acetonitrile and water with increasing percentage of water by volume from 0% to 90% (C = 1 × 10^−5^ mol/L, 20°C). (c) Partial ^1^H NMR spectra of CN‐OX‐COOMe in (i) CD_3_OD, (ii) CD_3_OD with TFA‐*d* (1.5 equiv.) and (iii) CD_3_OD/D_2_O (2:3, v/v). (d) Comparison of L^*^ curves between CN‐HSP‐10% PEG and CN‐OX&HSP‐10% PEG after response in different humidity environments. (e) Schematic illustration of the difference in the interaction of hydroxyl group and CN‐OX units in CN‐HSP and CN‐OX&HSP. (f) Optical microscope images of CN‐HSP and CN‐OX&HSP.

Subsequently, the molecular reaction mechanism of CN‐HSP‐10% PEG visual warning FFHUL was discussed in detail. After the introduction of water, CN‐HSP‐10% PEG rapidly transitions from colorless to purple, accompanied by a significant decrease in reflectance at 578 nm in the visible region of the ultraviolet‐visible (UV–vis) reflection spectrum (Figure 4a), confirming that the observed color change of CN‐HSP‐10% PEG is induced by water or humidity. To further verify this hypothesis and overcome the insolubility of CN‐HSP, a control molecule (CN‐OX‐COOMe) was synthesized by esterifying the carboxyl group of CN‐OX‐COOH with methanol. Its acetonitrile solution was initially colorless and turned purple after the introduction of water, and a new absorption peak centered at 576 nm appeared within the visible region of the UV–vis absorption spectrum, and its absorption intensity gradually increased with the increase of water content (Figure 4b), indicating that CN‐OX‐COOMe underwent water‐induced ring‐opening isomerization. The water‐induced structural conversion of CN‐OX‐COOMe was further confirmed by proton nuclear magnetic resonance (^1^H NMR) spectroscopy (Figure 4c). In deuterated ethanol (CD_3_OD), CN‐OX‐COOMe predominantly exists in its RCF (i). Upon the addition of deuterium water (D_2_O), a new set of proton signals (*a‐g*) emerged, and all of them shifted to the lower field (iii vs. i), similar to the signals after the addition of acid (iii vs. ii), which confirms the conversion of CN‐OX‐COOMe into its ROF zwitterionic isomer [38]. These experiments fully show that the color change of CN‐HSP‐10% PEG is owing to the water‐induced ring opening reaction of CN‐OX units.

The spatially fixed, multi‐level, and polyhydroxy‐sensitized microenvironment created by chemical grafting of CN‐OX onto cellulose elementary fibrils is crucial for the CN‐HSP‐10% PEG response to FFHUL‐100 ppm_v_. As shown in Figure 4d, chemically grafted CN‐HSP‐10% PEG showed a significant decrease in *L* ^*^ value when the humidity reached 100 ppm_v_. In stark contrast, CN‐ OX&HSP‐10% PEG, prepared by the physical blending with the same loading ratio of the sample synthesized by chemical grafting (Figure S3 and Equation S4), displayed a slight change only when the humidity was as high as to 3000 ppm_v_. This striking difference can be attributed to the stable and homogeneous covalently anchoring of CN‐OX within the cellulose elementary fibrils, which effectively disrupts the original strong hydrogen‐ bonding network among cellulose hydroxyl groups, so that the CN‐OX unit can be sensitized by a large number of free hydroxyl groups. Meanwhile, the problem of molecular aggregation is avoided, so that greatly improved the humidity sensitivity of CN‐HSP‐10% PEG. For CN‐OX&HSP‐10% PEG prepared by the physical blending method, most CN‐OX could only stay at the periphery of the microfibril bundles. Even for the few CN‐OX molecules that manage to penetrate the interior of the microfibril bundles, their sensitivity to humidity remains limited for two reasons: (i) they difficulty in breaking the strong intrinsic hydrogen‐bonding interactions among cellulose hydroxyl groups, leaving them exposed to only finite number of free hydroxyl groups for sensitization; and (ii) the strong *π*–*π* interactions between CN‐OX molecules promote self‐aggregation, further diminishing their humidity responsiveness (Figure 4e). This can be further confirmed by optical microscopy observations of a representative single fiber (Figure 4f), CN‐OX units were uniformly distributed and penetrate the interior of the microfibril bundles in CN‐HSP‐10% PEG, while CN‐OX&HSP‐10% PEG showed a clear aggregation phenomenon and surface enrichment.

**FIGURE 5:**
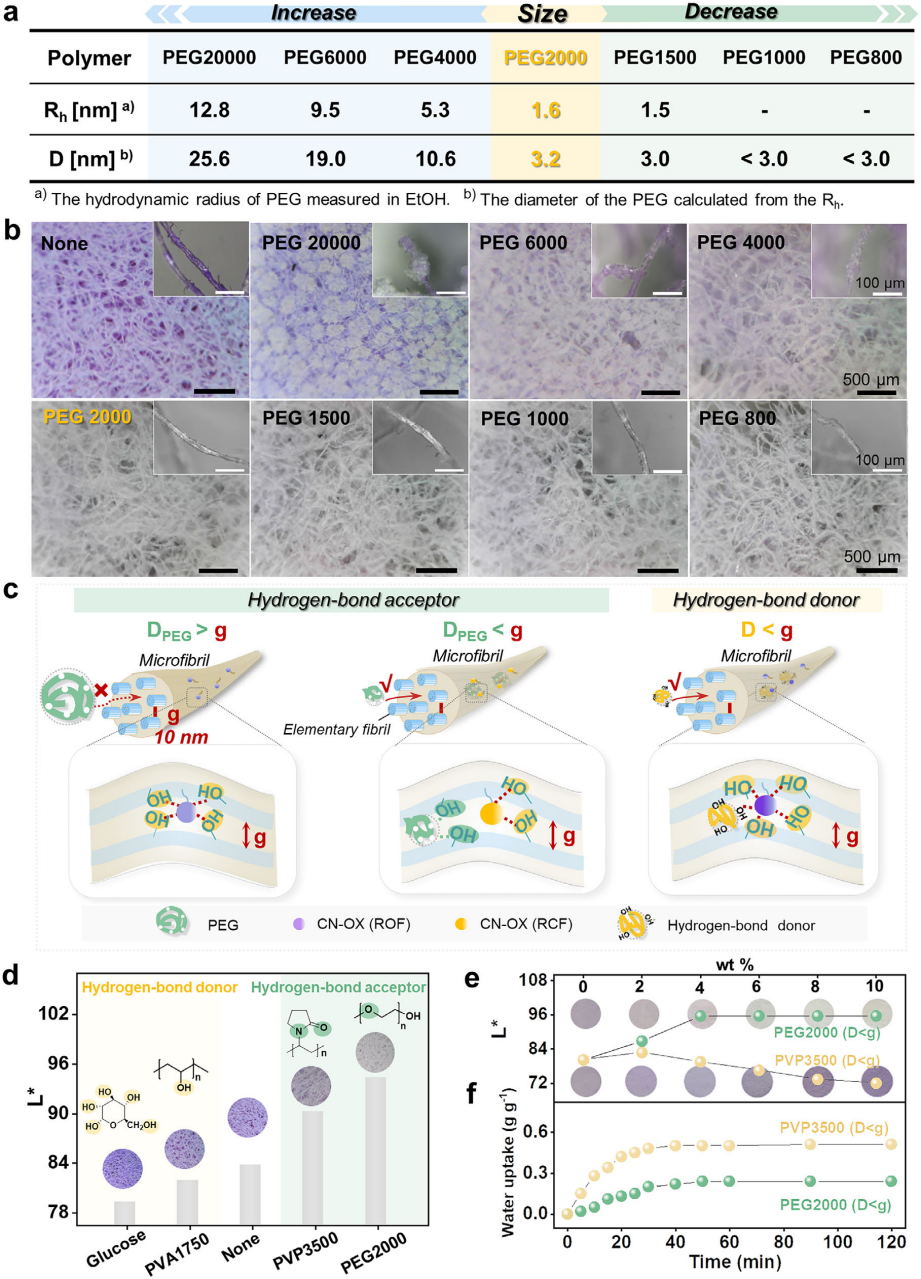
Compatibility of Size, Structure, and Functionality of the auxiliary medium. (a) Size of PEG with different molecular weights. (b) Optical microscope images of the initial state of CN‐HSP‐PEG after introducing PEG of different sizes. (c) Schematic diagram illustrating the microscopic interactions of CN‐OX units in CN‐HSP with differently sized PEG (left) and various hydrogen‐bond donors and acceptors (right). (d) Changes in the initial state color L^*^ value of CN‐HSP and corresponding optical microscope images after introducing different hydrogen‐bond donors (Glucose and PVA1750) and hydrogen‐bond acceptors (PVP3500 and PEG2000). (e) Changes in the initial state color L^*^ value of CN‐HSP and corresponding optical photos after introducing different amounts of PVP3500 and PEG2000. (f) Relative mass changes over time for PVP3500 and PEG2000 in a 20°C, 56% RH environment.

Moreover, the suitable size, hydrogen‐bond acceptor capability, and appropriate hygroscopic property of PEG2000 play vital roles in regulating the high‐contrast and accurate response of CN‐HSP‐10% PEG to the preset humidity threshold‐100 ppm_v_. First, based on the positive correlation between PEG molecular weight and size, we selected three types of PEGs with molecular weights progressively larger than PEG2000 (PEG4000, PEG6000, and PEG20000) and three with molecular weights gradually smaller than PEG2000 (PEG1500, PEG1000, and PEG800) to verify the significance of the optimal size of PEG2000 (Figure 5a; Figure S4 and Equation S1). These PEGs were infiltrated into the deep‐purple CN‐HSP using an identical method and mass ratio to fabricate a series of CN‐HSP‐PEGs. The initial coloration of these materials was observed to assess their “passivation” effectiveness. As shown in Figure 5b, the experimental results reveal that CN‐HSP‐PEGs obtained with larger PEGs (PEG20000, PEG6000, and PEG4000) exhibit a slightly lighter color compared to untreated CN‐HSP; but a distinct purple color remains visible within the microfibril bundles network. While the CN‐HSP‐PEGs prepared with smaller PEGs (PEG1500, PEG1000, and PEG800) display a completely colorless appearance, closely resembling the CN‐HSP‐PEG2000 (i.e., CN‐HSP‐10% PEG). This phenomenon can be observed more clearly in the magnified image of individual microfibril bundle (Figure 5b, inset). There are obviously colorless crystals on the surface of the single microfibril bundle of CN‐HSP‐PEGs obtained by PEG20000, PEG6000 and PEG4000 treatment, and the purple color inside the microfibril bundles is clearly visible, which indicates that large‐sized PEG fails to penetrate into the microfibril bundle interior, thereby the CN‐OX unit inside is still in ROF state. In contrast, CN‐HSP‐PEGs prepared with PEG2000, PEG1500, PEG1000, and PEG800 display a completely colorless state from the outer surface to the microfibril bundle interior, without the observation of PEG crystallites on the surface, which suggests that the small‐size PEGs can penetrate into the interior of the microfibril bundle, effectively passivating the CN‐OX units and inducing their transition from the purple ROF to the colorless RCF. Intriguingly, the PEGs capable of penetrating the microfibril bundles interior possess smaller sizes below 10 nm (Figure 5a), aligning well with the interstitial gap size (*g*, approximately 10 nm) between elementary fibrils within the microfibrils of microfiber bundles as reported in the literature [33, 34, 35, 36]. This finding not only further verifies the great size matching between PEG2000 and the pore structure of sensitized cellulose, but also provides guidance for understanding the size of the polymer and the microscopic mechanism of its interaction with cellulose, particularly in the research areas of polymer infiltration and functionalization.

Additionally, the hydrogen‐bond acceptor property of PEG2000 is crucial for adjusting the humidity response threshold of CN‐HSP‐PEG. Since PEG2000 can penetrate the interfibrillar gaps of the microfibrils, its abundant oxygen atoms can form hydrogen bonds with hydroxyl groups on the elementary fibril surface around the CN‐OX units. This above‐mentioned interaction effectively reduces the number of sensitizing hydroxyl groups surrounding CN‐OX units, thereby tuning the humidity response threshold of CN‐HSP‐PEG (Figure 5c, left). In order to verify this mechanism, polyvinyl pyrrolidone with a molecular weight of 3500 (PVP3500), also as a hydrogen‐bond acceptor with a size smaller than *g*, was performed. Compared to untreated CN‐HSP, the infiltration of PVP3500 resulted in a significant lightening or near‐complete fading of the purple color, along with a corresponding increase in L^*^ value—similar to the effect observed with PEG2000. In contrast, introducing polyvinyl alcohol (PVA1750) and glucose, both of which are hydrogen‐bond donors and also smaller than *g*, lead to a deepening of the purple color and a decrease in L^*^ value (Figure 5d). This effect was particularly evident with glucose, which contains more hydroxyl groups than PVA1750, causing to an even deeper color change. These experimental results demonstrate that the introduction of hydrogen‐bond donors increases the number of hydroxyl groups surrounding CN‐OX units, which will further sensitize the ring‐opening isomerization of CN‐OX and further increase/deepen its ROF ratio/color (Figure 5c, right). Conversely, introducing hydrogen‐bond acceptors effectively reduces the hydroxyl concentration around CN‐OX units, passivating the above ring‐opening isomerization. This modulation significantly elevates the humidity response threshold and stabilizes the initial state of CN‐HSP‐PEG in a colorless OFF state. Notably, the ideal passivating effect observed with PEG2000 cannot be achieved using PVP3500, despite both being hydrogen‐bond acceptors. As shown in Figure 5e, an increase in PEG2000 content causes the initial color of CN‐HSP‐PEG gradually fades to a colorless state, with the corresponding L^*^ value rising and stabilizing to 96. In contrast, under the same conditions, increasing the PVP3500 content initially causes a slight color lightening of CN‐HSP‐PVP, followed by a deepening change, with the corresponding L^*^ value rise slightly and then fall gradually. This phenomenon is due to the fact that PVP3500 exhibits stronger hygroscopicity than PEG2000 and is able to absorb more water molecules (Figure 5f), thereby more effectively promoting the ring‐opening isomerization of CN‐OX than the passivating effect caused by the hydrogen‐bond acceptor property of PVP3500. This finding further highlights the crucial role of the suitable hygroscopicity for PEG2000 in maintaining CN‐OX in the ROF state under initial conditions and in modulating its response to a specific humidity response threshold.

**FIGURE 6:**
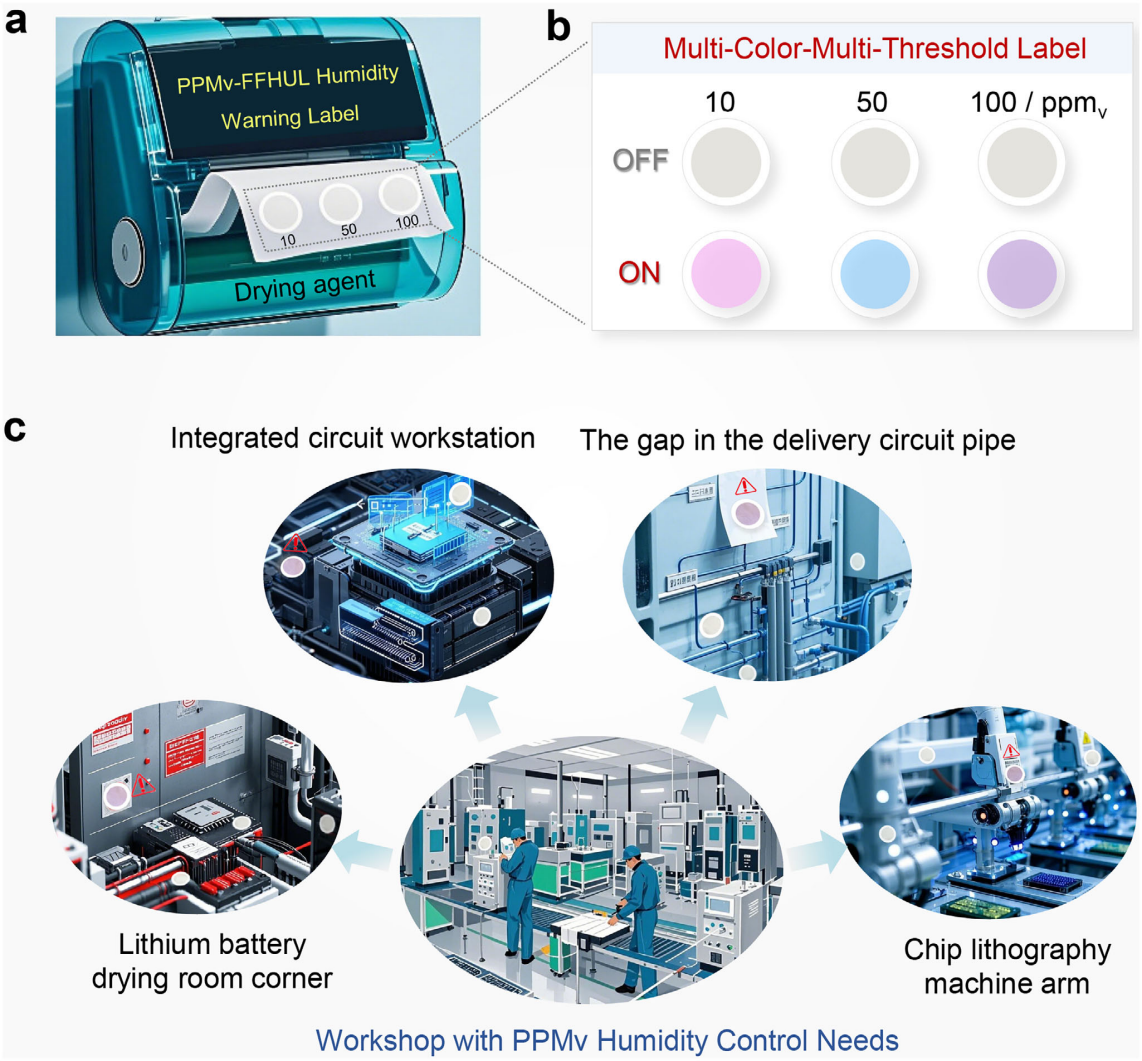
Application prospects. (a) Schematic diagram of the structure of the portable ppm_v_‐FFHUL humidity warning label. (b) Schematic diagram of the Multi‐Color‐Multi‐Threshold Label designed using hydrochromic molecular switches of different colors. (c) Schematic diagram of the large‐scale, multi‐point deployment of ppm_v_‐FFHUL humidity warning label within the confined production environments of various critical manufacturing sectors.

In principle, our strategy described herein should permit us to achieve a “multi‐color‐multi‐threshold” portable ppm_v_‐FFHUL humidity warning label by designing and selecting hydrochromic molecular switches with different colors and sensitivities, along with their matched number of spatially fixed polyhydroxy microenvironments (Figure 6a) Prior to use, the warning label is kept in an off state under the protection of desiccant (calcium oxide). During usage, users can remove the package and expose the multi‐colored strips or single‐color area to the test environment (Figure 6b). By simply observation of the color changes with the naked‐eye, users can conveniently and quickly determine/forecast whether the current environmental humidity poses a threat to devices/equipment. The portable ppm_v_ humidity label features several advantages such as high sensitivity, portability, and cost‐effectiveness, which make it as an ideal choice for humidity monitoring and early warning in the field of ppm_v_‐level humidity environments in the future. For instance, it can be extensively applied in confined spaces such as integrated circuit operation workshops, gaps in transmission circuit pipelines, corners of lithium battery drying rooms, and operational arms of chip photolithography machines, enabling large‐scale deployment across multiple points [39] (Figure 6c), ensuring that humidity levels throughout the facility remain within monitorable ranges and allowing for real‐time monitoring and early warning of ppm_v_ humidity through naked‐eye observation of color changes. This approach is particularly effective in preventing issues of localized high humidity caused by inadequate air ventilation, thereby ensuring product quality and production efficiency.

## 3 Conclusions

In summary, this study has achieved rapid reading of specified ppm_v_‐level FFHUL by only the naked‐eye for the first time by precisely constructing a sensitized microenvironment with a matching number of spatially fixed, multi‐level hydroxyl sensitizing functional groups around an elaborately designed humidity‐sensitive component (CN‐OX) at the molecular level in an “enzyme‐like” manner. The obtained material (i.e., CN‐HSP‐10% PEG) features high naked‐eye recognition contrast (*ΔE* = 12, far exceeding the human eye recognition threshold *ΔE* = 4), fast response speed (only within 5 min), and effective early‐warning capability (20% prior to FFHUL). The underlying mechanism of this visualization is attributed to the rationally designed ring‐opening isomerization reaction of CN‐OX, where the design of cyano groups has been proven to play a crucial role in achieving both high contrast and rapid response. The spatially fixed, multi‐level polyhydroxy sensitized microenvironment formed by chemically bonding multi‐level sensitizing matrix was confirmed as a key factor enabling ppm_v_‐level humidity visualization. Besides, the size‐matched supramolecular interaction between the passivator PEG2000 and the sensitizing matrix enabled facile and precise regulation of the number of active hydroxyl groups on demand, and together with its appropriate hygroscopicity, contributed to the “leap change” response behavior of CN‐HSP‐10% PEG toward specific ppm_v_‐level humidity. The relevant mechanisms have been systematically validated through various characterization techniques, including ultraviolet‐visible spectroscopy infrared spectroscopy, proton nuclear magnetic resonance, and optical microscopy. The as‐prepared material combines paper‐like flexibility, facile fabrication and handling, and enables high‐contrast readout of the target ppm_v_‐FFHUL only by naked‐eye. Although the current detection accuracy of this technology is not comparable to commercial precision detection equipment, similar to the distinct positioning of pH test papers and pH meters, this “test paper‐like” detection technology, with its unique advantages of low cost and portability (Table S3), holds substantial application value in specific scenarios that require early warning and on‐site rapid screening of ppm_v_‐level humidity. This study can not only greatly promote the development of visual ultra‐low humidity sensing. More importantly, the proposed “enzyme‐like” precise construction concept and “leap change” response mode also provide innovative ideas for the portable and visual sensing of other trace gases/components. The “polymer size—cellulose pore size” matching law revealed in this study provides a new perspective for evaluating polymer size and studying their interfacial microscopic interaction mechanisms with celluloses.

## Conflicts of Interest

The authors declare no conflicts of interest.

## Supporting information




**Supporting File**: advs73547‐sup‐0001‐SuppMat.pdf.

## Data Availability

The data that support the findings of this study are available from the corresponding author upon reasonable request.
