# Supplementary material for: Naked‐Eye Visual Warning of ppmv‐Level Fault‐Free Humidity Upper Limit
Source: Adv Sci (Weinh). 2026 Jan 29;13(14):e18311. doi: 10.1002/advs.202518311 (PMC12970268; doi:10.1002/advs.202518311)
Supplement: Supplementary file 1 — Supporting File: advs73547‐sup‐0001‐SuppMat.pdf. [file ADVS-13-e18311-s001.pdf]

WILEY-VCH

Supporting Information

Naked-eye Visual Warning of ppm_v-level Fault-free Humidity Upper Limit

Xiaoyan Wei[a], Tianyou Qin[b], Siyu Wang[a], Sean Xiao-An Zhang[a] and Lan Sheng[a,*]

[a]State Key Lab of Supramolecular Structure and Materials, College of Chemistry, Jilin University, Changchun 130012, P. R. China

[b]Department of Biochemistry and Molecular Biology, College of Basic Medicine Science, Jilin University, Changchun 130012, P. R. China

*Corresponding Author. Email: shenglan17@jlu.edu.cn.

Table of contents:

1. Experimental section........S-2
2. Synthesis and characterization........S-6
3. Estimation of the mean square radius of gyration (R_g) of PEG........S-10
4. Determination of degree of substitution (DS)........S-11
5. The effect of PEG introduction method and content on CN-HSP-PEG........S-12
6. Gas selectivity test........S-13
7. Calculation of color difference values (ΔE)........S-14
8. Stability and repeatability tests........S-15
9. Calculation of molecular switch load ratio........S-16
10. Measurement of the hydrodynamic radius (R_h) of PEG........S-18
11. Performance comparison........S-19
12. References........S-20

1. Experimental Section

Materials.

Synthetic raw materials: 4-cyanophenylhydrazine hydrochloride (98%), 3-methyl-2-butanone (98%), 4-dimethylaminopyridine (DMAP, 98%), dicyclohexylcarbodiimide (DCC, 98%), anhydrous phosphorus oxychloride (99.5%), and anhydrous N, N-dimethylformamide (DMF), were purchased from Energy Chemical (Shanghai, China). N-ethyl-N-cyanoethylaniline (98%), 2-bromoethanol (95%), were purchased from Aladdin Co., Ltd. (Shanghai, China). Unless otherwise noted, all the other materials were purchased from Energy Chemical and were used without further purification.

Polymers: Polyethylene glycol (PEG) with different molecular weights- PEG 20000, PEG6000, and PEG2000 were obtained from Huadong Reagent Factory (Tianjin, China). PEG4000, PEG1500, PEG1000 and PEG800 were acquired from Energy Chemical (Shanghai, China). Polyvinylpyrrolidone with a molecular weight of 3500 (PVP3500) was purchased from Energy Chemical (Shanghai, China). Polyvinyl alcohol with a molecular weight of 1750 (PVA1750) was obtained from Aladdin Co., Ltd. (Shanghai, China), and glucose was sourced from Beijing Chemical Reagent Company (Beijing, China).

Salts: Calcium oxide (CaO) was purchased from Guangfu Fine Chemical Research Institute (Tianjin, China). Anhydrous sodium sulfate (Na_2SO_4) and sodium bicarbonate ($NaHCO_3$) were obtained from Beijing Chemical Reagent Company (Beijing, China).

Solvents: Tetrahydrofuran (THF), acetonitrile (MeCN), Dichloromethane (DCM), anhydrous ethanol (EtOH), and methanol (MeOH) were purchased from Yuwang Group (Shandong, China). Triethylamine (Et_3N) was purchased from Xilong Chemical Reagent Company (Guangdong, China). Ethyl acetate (EtOAc) was sourced from Beijing Chemical Reagent Company (Beijing, China).

Other reagents: Deionized water was obtained by purifying commercially available Wahaha water (Hangzhou, China) with the PALL PURELAB Plus instrument. Hydrochloric acid (HCl), concentrated sulfuric acid (H_2SO_4) and glacial acetic acid (CH_3COOH) were purchased from Beijing Chemical Factory (Beijing, China). Cellulose filter paper was purchased from Waterman-Xinhua Company (Hangzhou, China).

Instruments.

^{1}H NMR (400 MHz) and ^{13}C NMR (101 MHz) spectra were recorded at room temperature using an AS 400 spectrometer from Zhongke Oxford Instrument Company. Chemical shift values for protons were referenced to the residual proton resonance of DMSO-d6 (δ: 2.50 ppm). Chemical shift values for carbons were referenced to the resonances of DMSO (δ: 39.52 ppm). LC-HRMS analysis was carried out on an Agilent 1290-micro TOF-QII mass spectrometer. FT-IR spectra were recorded on Vertex 80 / 80V FT-IR spectrometer with LNMCT Mid DC detector over the range of 4000-800 cm^{-1} using KBr as reference. Elemental analysis was conducted using a Vario EL/micro cube elemental analyzer (Germany). The hydrodynamic radius of PEG was determined using a Nano Particle Size and Zeta Potential Analyzer (dynamic light scattering, DLS) from Malvern Panalytical Ltd. (UK). Ultraviolet-visible (UV-vis) absorption and reflectance spectra were collected by Analitik Jena Specord®210 plus UV/Vis spectrophotometer. The latter was tested via reflective mode of integrating sphere using $BaSO_4$ powder as background. The *L*a*b** color values of the samples were determined using an X-Rite spectrodensitometer. Photographs were collected by Nikon D7100 cameras. A custom-designed Vigor glove box (Vigor, Suzhou, China) was employed to create controlled humidity environments with different ppm_v levels.

Experimental operations

Preparation of cellulose microfiber

Soak the filter paper scraps in EtOH and stir at high speed until it forms a slurry, then filter. The obtained filter cake is repeatedly washed with EtOH and THF, after which the solvent is removed in an oven at 85 °C to obtain the pretreated microfibers.

Preparation and Storage of CN-HSP

To prepare CN-HSP, 0.25 g of CN-OX-Fiber was dispersed in 30 mL of EtOH under vigorous stirring, ensuring uniform dispersion. The mixture was then filtered under an inert atmosphere of argon or nitrogen to remove the solvent. Subsequently, the obtained material was cut into various sizes and shapes as required. The prepared CN-HSP samples were then stored in a sealed environment above solid calcium oxide powder for further use.

Preparation and Storage of CN-HSP-PEG

To prevent the loss of PEG during the material forming process, PEG was quantitatively and controllably dissolved in a specific volume of EtOH, a polar solvent compatible with the highly polar environment of cellulose. The fibers were then allowed to autonomously absorb the PEG solution through capillary action, integrating it into their internal structure.

Creation of different ppm_v humidity environments

By introducing a small amount of water into the glove box (add water droplets to the toilet paper and then shake back and forth in the glove box) or turn on the cyclic dehumidification mode to increase or decrease the humidity inside the box, and then use the wind power of the fan to evenly distribute the humidity atmosphere inside the box to create a humidity environment of different ppm_v levels.

Hygroscopicity test

The hygroscopicity of the polymer is evaluated using a controlled temperature and humidity test chamber. The testing process is described using a PEG film as an example. Dissolve 200 mg of PEG powder) in 5 mL of DCM, then transfer a small amount of this solution into a polytetrafluoroethylene mold (2 cm in diameter, 0.5 cm deep). After the DCM naturally evaporates, a PEG film is obtained. The PEG film is then placed in the pre-set and stabilized temperature and humidity chamber, where its mass is monitored over time using a precision balance to evaluate its hygroscopic properties.

2. Synthesis and characterization

Synthesis of CN-OX-COOH

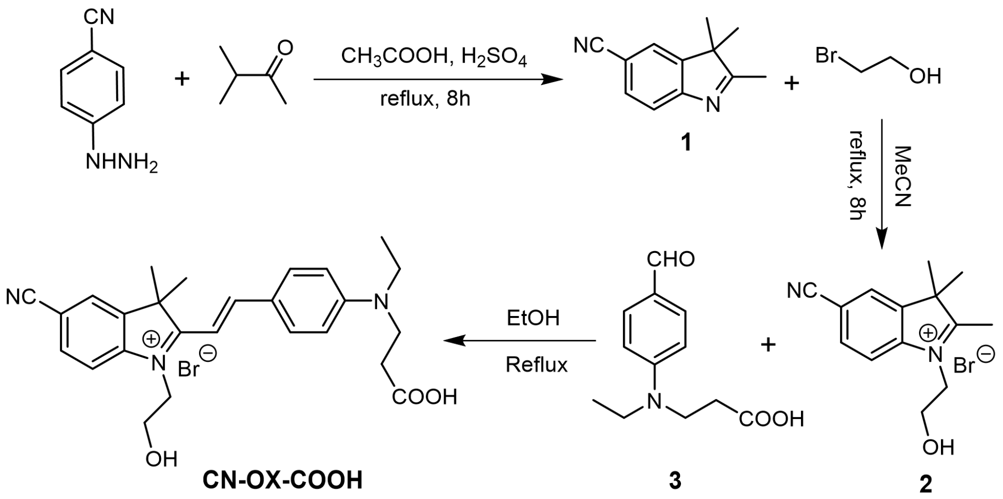

Scheme S1. Synthetic route of CN-OX-COOH.

Reactants of 3-(ethyl(4-formylphenyl) amino) propanoic acid (compound **3**) was synthesized according to literature procedures[S1] and its structural characterization data were consistent with the reported values.

2,3,3-trimethyl-3H-indole-5-carbonitrile (compound 1): 3.24 g (20 mmol) 4-hydrazineylbenzonitrile, 1.3 g (26 mmol) 3-methylbutan-2-one and 5 mL of H_2SO_4 were dissolved in 20 mL CH_3COOH and refluxed under a nitrogen atmosphere for 8 hours. Upon completion of the reaction, as confirmed by thin-layer chromatography (TLC), heating was stopped, and the reaction mixture was cooled to room temperature. The majority of the solvent was then removed under reduced pressure via distillation. The residual solution was diluted with an appropriate amount of deionized water and subsequently neutralized with a 10% aqueous Na_2CO_3 solution. The mixture was extracted with EtOAc (3×50 mL), and the combined organic layers were dried over anhydrous Na_2SO_4. The solvent was removed under reduced pressure, and the crude product was obtained by filtration. The final product, a white powder (compound 1), was purified via column chromatography (SiO_2; DCM/CH_3OH, 60:1, v/v) (yield 64%).

^{1}H NMR (400 MHz, DMSO-d_6): δ 7.99 (s, 1H), 7.78 (d, J = 8.0 Hz, 1H), 7.61 (d, J = 8.0 Hz, 1H), 2.27 (s, 3H), 1.28 (s, 6H).

1-(2-hydroxyethyl)-2,3,3-trimethyl-5-cyano-3H-indol-1-ium (compound 2): 2 g (11.2 mmol) compound 1 and 1.6 ml (24 mmol) 2-bromoethanol were dissolved in 12 mL MeCN and refluxed under a nitrogen atmosphere for 24 hours. Upon completion of the reaction, as confirmed by TLC, the reaction mixture was cooled to room temperature, leading to the precipitation of a solid. A large volume of EtOAc was then added to induce further precipitation. The crude product was collected by filtration and subsequently washed multiple times with small portions of EtOAc to afford the target compound 2 as a purple-black solid powder (yield 76%). ^{1}H NMR (400 MHz, DMSO-d_6): δ 7.64-7.52 (m, 2H), 7.04 (d, J = 8.0 Hz, 1H), 5.48 (s, 1H), 3.84-3.72 (m, 2H), 3.62-3.50 (m, 2H), 1.27 (s, 6H), 1.12 (s, 3H).

2-(4-((2-carboxyethyl)(ethyl)amino)styryl)-5-cyano-1-(2-hydroxyethyl)-3,3-dimethyl-3H-indol-1-ium (CN-OX-COOH): 3.8 g (12 mmol) compound **2** and 3.3 g (15 mmol) compound **3** were dissolved in 18 mL EtOH and refluxed for 7 hours under N_2 protection. After cooling to room temperature, a large amount of solid precipitation was observed in the reaction solution, which was then filtered. The resulting solid was washed with EtOAc (15 mL) three times to obtain purple-black solid powder (yield 80%). ^{1}H NMR (400 MHz, DMSO-d_6): δ 12.46 (s, 1H), 8.43 (d, J = 16.0 Hz, 1H), 8.34 (s, 1H), 8.10 (d, J = 16.0 Hz, 2H), 8.03 (d, J = 8.0 Hz, 1H), 7.84 (d, J = 8.0, 1H), 7.32 (d, J = 16.0, 1H), 6.95 (d, J = 8.0 Hz, 2H), 5.12 (s, 1H), 4.58 (s, 2H), 3.83-3.75 (m, 4H), 3.64-3.60 (m, 2H), 2.70-2.58 (m, 2H), 1.79 (s, 6H), and 1.20-1.15 (m, 4H) ppm. ^{13}C NMR (101 MHz, DMSO-d_6): δ 182.16, 173.59, 172.04, 156.91, 154.40, 146.34, 144.12, 134.32, 127.63, 123.80, 119.68, 1145.64, 113.54, 109.50, 106.05, 61.47, 51.66, 48.90, 46.88, 46.02, 33.08, 27.45, 19.50, 15.01, 13.25 ppm. LC-HRMS (ESI) m/z calcd. for $C_{26}H_{30}BrN_3O_3$ $[M-Br]^{+}$: 432.2287; found 432.2285.

Synthesis of CN-OX-COOMe

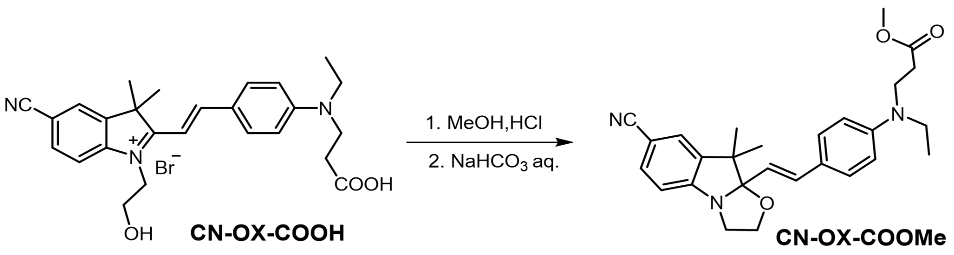

Scheme S2. Synthesis procedure of CN-OX-COOMe.

Methyl(E)-3-((4-(2-(7-cyano-9,9-dimethyl-2,3-dihydrooxazolo[3,2-a]indol-9a(9H)-yl)vinyl)phenyl)(ethyl)amino)propanoate (CN-OX-COOMe): 204 mg (0.4 mmol) CN-OX-COOH and 40 μL HCl (0.5 mmol) were dissolved in MeOH (8 mL). The solution was refluxed for 12 hours under nitrogen protection and concentrated under reduced pressure. Saturated $NaHCO_3$ solution (50 mL) was added and stirred at room temperature for 4 hours. At this time, the solution changes from the initial blue-purple to yellow almost completely. And the emulsion was extracted with EtOAc (3×20 mL), dried over Na_2SO_4, filtered and concentrated. The product was obtained as a yellow solid (yield 53%). ^{1}H NMR (400 MHz, DMSO-d_6): δ 7.61 (s, 2H), 7.35 (d, J = 8 Hz, 2H), 7.06 (d, J = 8.0 Hz, 1H), 6.66-6.62 (m, 3H), 6.02 (d, J = 16.0 Hz, 1H), 3.78-3.69 (m, 2H), 3.61-3.55 (m, 7H), 3.37-3.36 (m, 2H), 2.58-2.54 (t, J= 14.0 Hz, 2H), 1.37 (s, 3H), 1.08 (s, 3H) and 1.05 (t, J = 14.0 Hz, 3H) ppm. ^{13}C NMR (101 MHz, DMSO-d_6): δ 172.30, 155.13, 149.85, 134.62, 130.17, 130.02, 129.71, 129.14, 127.02, 125.90, 118.60, 114.59, 111.79, 111.69, 101.72, 64.52, 59.91, 51.90, 51.72, 48.16, 47.19, 33.28, 18.58, 12.85 ppm. LC-HRMS (ESI) m/z calcd. for $C_{27}H_{31}N_3O_3$ $[M+H]^+$: 446.2438; found 446.2433.

Synthesis of CN-OX-Fiber

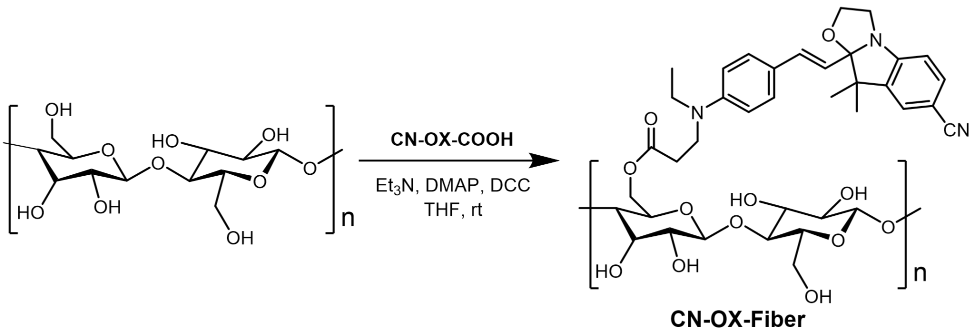

Scheme S3. Synthesis of CN-OX-Fiber.

CN-OX-Fiber: A mixture of 3 g of pretreated cellulose, 900 mg (1.7 mmol) of CN-OX-COOH, 200 mg (2 mmol) of Et_3N, and 210 mg (1.7 mmol) of DMAP was dissolved in 150 mL of THF and stirred thoroughly. Under ice-salt bath conditions, 1.05 g (5 mmol) of DCC dissolved in 16 mL of THF was slowly added dropwise to the reaction mixture. After the addition was complete, stirring was continued in the ice-salt bath for 2 hours, followed by reaction at room temperature for 24 hours. Upon completion, the reaction mixture was filtered under an inert atmosphere of argon or nitrogen. The solid was repeatedly washed with THF and EtOH to remove impurities and unreacted dye molecules. To ensure complete removal of unreacted CN-OX-COOH, a few drops of dilute HCl were added to the filtrate; if no purple color appeared, the washing was considered sufficient. The purified CN-OX-Fiber was then obtained and stored in ethanol for further use.

3. Estimation of the mean square radius of gyration (*Rg*) of PEG:

The *Rg* is a statistical parameter used to describe the spatial dimensions of a polymer chain in solution or solid state. It represents the mean squared distance of individual units (such as monomers or atoms) from the center of mass of the polymer chain. The calculation formula for the Rg of PEG is as follows [S2]:

$$R = \frac{1}{\sqrt{6}} \times \left(\frac{M}{137}\right)^{a} \times 1.1 \qquad \textbf{Equation (1)}$$

Here, *R* represents the Rg of PEG, measured in nanometers (nm); *M* denotes the molecular weight of PEG; and *a* is a constant, with a value of 0.588 in solution and bulk phase, while for high molecular weight PEG (M > 20000), the value in the bulk phase is 0.5.

4. Determination of degree of substitution (DS)

Table S1. Nitrogen content (*N%*) and DS of CN-OX-Fiber and Cellulose in different grafted batches.

Reaction batches [a]	N%		DS
	CN-OX-Fiber	Cellulose	
1st	0.72	0.06	0.03
2nd	0.68	0.04	0.03
3rd	0.69	0.04	0.03

[a] Different batches of grafting reactions.

The DS of CN-OX units on the Cellulose was calculated through the nitrogen content, according to following equation:

$$DS = \frac{M_{cellulose} \times N\%}{M_N - M_{CN-OX} \times N\%} \qquad \textbf{Equation (2)}$$

Where *N%* is the amount of nitrogen in CN-OX-Fiber in weight percentage, 162 g/mol is the molecular weight of anhydroglucose unit, M_N is the molar mass of nitrogen atoms that are grafted molecule (42 g/mol for the CN-OX), M_{CN-OX} is the molar mass of grafted dyes (432 g/mol for the CN-OX).

5. The effect of PEG introduction method and content on CN-HSP-PEG

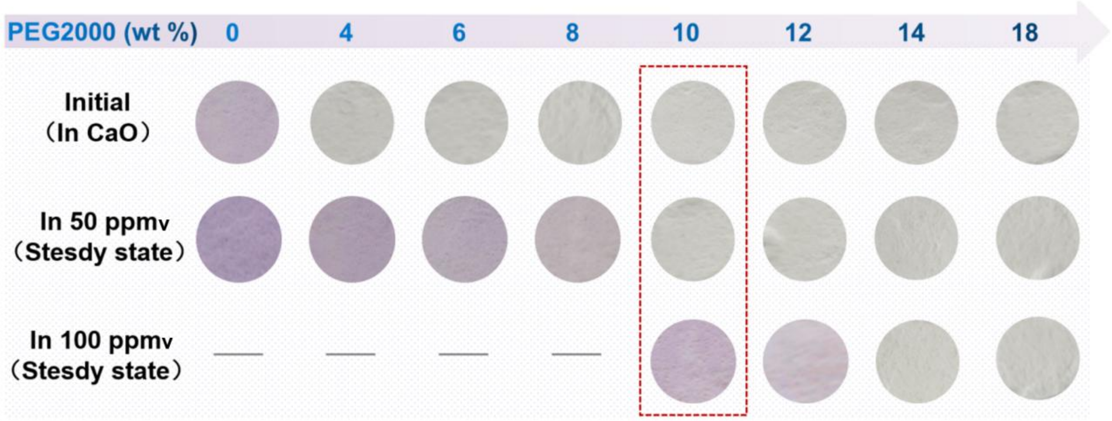

Figure S1. Photographs of CN-HSP-PEG with different PEG2000 contents before and after response under 50 and 100 ppm_v humidity conditions.

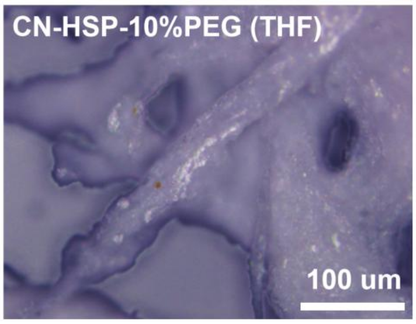

Figure S2. Optical microscope images of CN-HSP-10% PEG constructed using THF as a permeation solvent.

6. Gas selectivity test

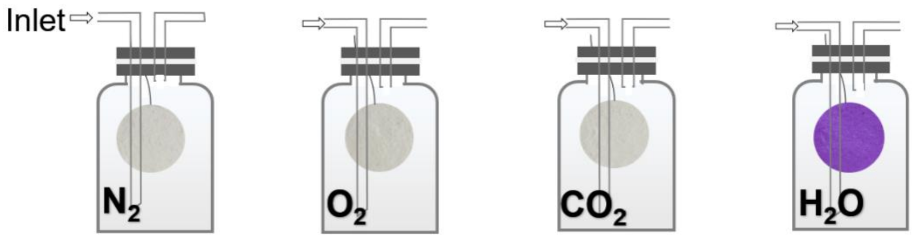

Figure S3. Photographs of CN-HSP-PEG placed in different gas atmospheres.

7. Calculation of color difference values (*ΔE*)

The *CIE Lab color space* was proposed by the International Commission on Illumination. In the CIE Lab space, any color in nature can be represented, and it aligns well with human visual perception. It is currently the most comprehensive and uniform color model used to describe all colors visible to the human eye. The Lab color model consists of three components:

L^* represents lightness, ranging from 0 (black) to 100 (brightest white); a^* indicates the transition from green to red, with negative values representing green and positive values representing red; b^* denotes the transition from blue to yellow, with negative values representing blue and positive values representing yellow.

In this uniform color space, the distance between two color coordinate points corresponds to the ΔE between two color samples. The formula for calculating ΔE is as follows:

$$\Delta E = \sqrt{\Delta L^2 + \Delta a^2 + \Delta b^2}$$ **Equation (3)**

Here, ΔL, Δa, and Δb represent the differences in L^*, a^*, and b^* values between two colors, respectively.

Table S2. Correspondence between Δ*E* and human visual perception.

**ΔE**	**< 4**	**4-6**	**6-12**	**> 12**
Human visual perception	indistinguishable	perceptible	noticeable	intense

8. Stability and repeatability tests

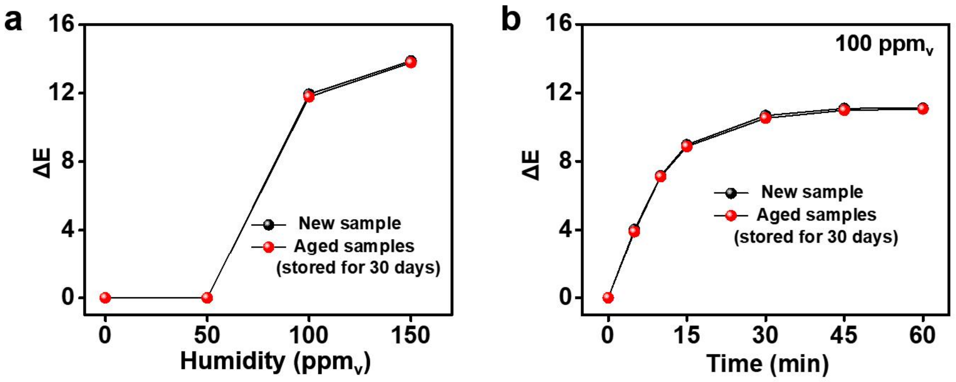

Figure S4. (a) Comparison of color changes after response under different ppm$_v$ humidity conditions between the new sample and the aged sample stored for 30 days and (b) comparison of response rates under 100 ppm$_v$ humidity condition.

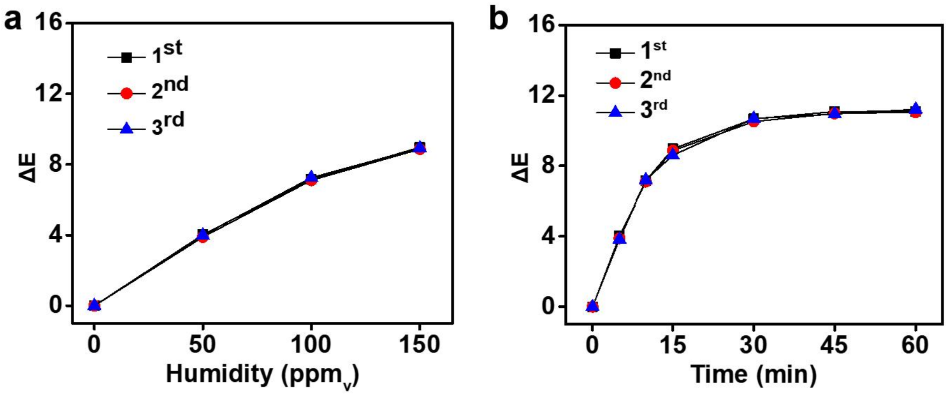

Figure S5. (a) Comparison of color changes of samples prepared in different batches after response under different ppm$_v$ humidity conditions; (b) comparison of response rates of them under 100 ppm$_v$ humidity condition.

9. Calculation of molecular switch load ratio

We use the method of equivalent of concentration to measure the concentration of unreacted CN-OX-COOH molecules in the reaction filtrate to calculate the number of CN-OX-COOH molecules involved in the grafting reaction. First of all, five different concentrations of standard solutions of CN-OX-COOH were prepared, and their UV-vis absorption spectra were measured, then the curves of absorbance at their λ_{max} versus concentrations were plotted (A-C curves). Next, add excess HCl to the reaction filtrate (make sure CN-OX-COOH molecule is fully ring-opening), and the obtained solutions were diluted a certain multiple with the corresponding solvent to make the absorbance (λ_{max}) within the absorbance range of the standard solutions. The concentration of unreacted CN-OX-COOH molecules in the reaction filtrate determined based on their absorbance (λ_{max}) and A-C curves.

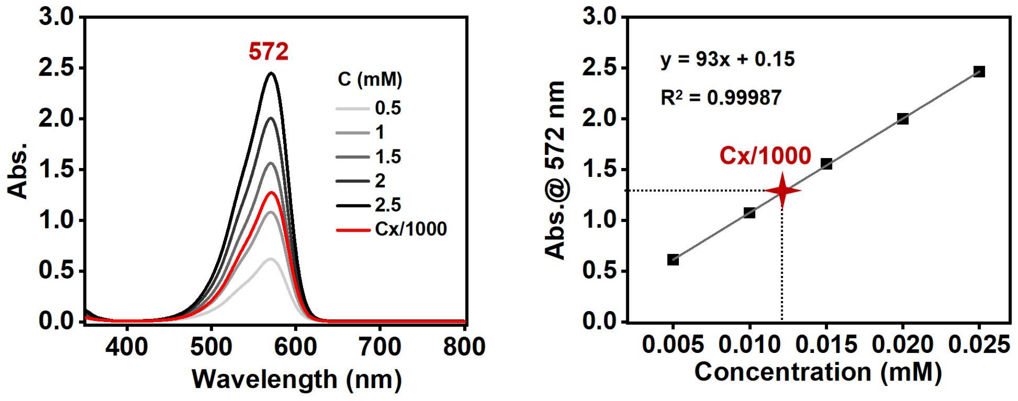

Figure S6. UV-vis absorption spectra of CN-OX-COOH with different concentrations in EtOH (left) and the plot of the absorbance at 572 nm (right), inset is the regression equation of this standard curve.

The molecular switch load ratio (L) was determined from following equation:

$$L = \frac{W_{graft\ dyes}}{W_{Cellulose}} = \frac{W_{initial\ dyes} - C_X \cdot V \cdot M_{dyes}}{W_{Cellulose}} \qquad \textbf{Equation (4)}$$

Where L is the CN-OX weight grafted on per gram of cellulose, mg/g; W is the weight of cellulose or CN-OX-COOH, mg; C_X is the concentration of unreacted

CN-OX-COOH molecules in the filtrate, mol/L; M_{dyes} is the molar mass of the CN-OX-COOH, g/mol; V is the total volume of the filtrate, mL.

10. Measurement of the hydrodynamic radius (R_h) of PEG

Due to the fact that some PEG samples have sizes too small to be characterized by conventional static light scattering instruments designed for measuring the R_g (these instruments typically have a size range above 10 nm), DLS was employed in this study to characterize the R_h of PEG in solution, thereby assessing the size variations of PEG molecules with different molecular weights. The measured R_h were then converted to R_g values using the Kirkwood-Riseman theoretical formula (S3). The calculated R_g values obtained through this method are largely consistent with those computed directly from the radius of gyration formula. For PEG1000 and PEG800, their R_h were too small to be measured with the available techniques.

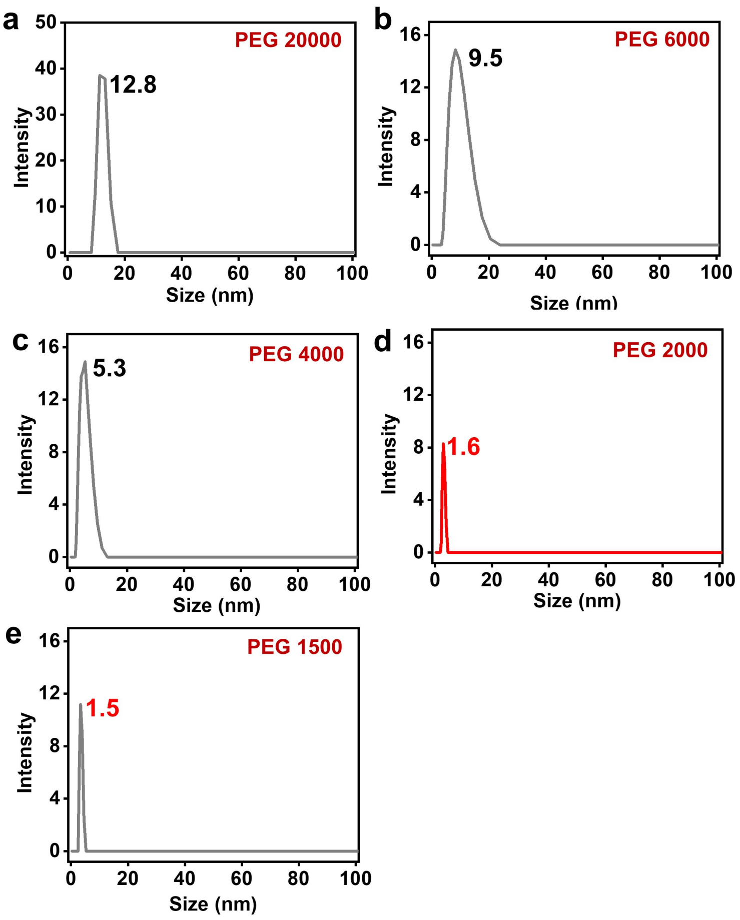

Figure S7. The R_h sizes of PEGs with different molecular weights measured by DLS, a) PEG20000; b) PEG6000; c) PEG4000; d) PEG2000; e) PEG1500 (in EtOH solvent).

11. Performance comparison

Table S3. Performance comparison between the technology reported in this work and other existing technologies.

	**Electronic humidity sensor**	**Moisture dew-point meters**	***This work***
Detection limit	0.01-100 ppm_v	0.01-100 ppm_v	100 ppm_v
Response time	< 1 min	< 1 min	> 5 min
Signal form	Electrical signal	Temperature	Color
Sensor volume	Bulky (> 3000 cm^3)	Bulky (> 5000 cm^3)	Paper-like (Cut as needed)
Portability	Poor	Poor	Good
Purchase cost	> $2500	> $4000	< $5
Applicable scenarios	Continuous and precise detection	Continuous and precise detection	Rapid screening by naked-eye

12. References

[S1] X. Wei, S. X.-A. Zhang, L. Sheng, "Enzyme-like" spatially fixed polyhydroxyl microenvironment-activated hydrochromic molecular switching for naked eye detection of ppm level humidity. *Adv. Mater*. **2023**, 35, 2208261.

[S2] Rubinstein, Michael, H. Colby. Ralph, *Polymer Physics*. Oxford University Press, **2003**.

[S3] J. G. Kirkwood, J. Riseman, The Intrinsic Viscosities and Diffusion Constants of Flexible Macromolecules in Solution. *J. Chem. Phys.* **1948**, 16, 565.
